# Intracranial bullet removal using O-arm navigation guidance

**DOI:** 10.1007/s00381-020-05028-0

**Published:** 2021-01-22

**Authors:** Tuija Keinänen, Maija Lahtinen, Susanna Piironen, Juha-Jaakko Sinikumpu, Niina Salokorpi, Jani Katisko

**Affiliations:** 1 Department of Surgery, Operative Care Unit, Oulu University Hospital, PO-BOX 21, 90029 OYS Oulu, Finland; 2 Oulu Research Group of Advanced Surgical Technologies and Physics (ORGASTP), Medical Research Center Oulu (MRC Oulu), Oulu University Hospital and University of Oulu, Oulu, Finland; 3 PEDEGO Research Group and MRC Oulu, University of Oulu, Oulu, Finland; 4 Department of Children and Adolescents, Oulu University Hospital, Oulu, Finland; 5 Research Unit of Clinical Neuroscience, Medical Research Center Oulu (MRC Oulu), Oulu University Hospital and University of Oulu, Oulu, Finland

**Keywords:** Neurosurgery, Intraoperative imaging, Navigation, Foreign body

## Abstract

**Purpose:**

The purpose of this study is to report a new mini-invasive technique to remove an intracranial bullet in a child by using O-arm for intraoperative neuronavigation.

**Case report:**

A 14-year-old refugee boy had suffered a shooting injury 4 years earlier. O-arm imaging-assisted neuronavigation during craniotomy was performed in order to remove a bullet from the intracranial space in a paediatric patient.

**Conclusion:**

Navigation using O-arm is a feasible method in removing a foreign material in a child and gave an accurate location of the bullet in the adopted surgical operation position without significant imaging artefacts.

## Introduction

Image-guided surgery and neuronavigation is used routinely when an accurate localization and demarcation of region of surgical interest (ROSI) is required. Commonly, neuronavigation is based on preoperatively obtained magnetic resonance imaging (MRI) or computed tomography (CT) images. MRI has the advantage of good spatial resolution with high soft tissue contrast. However, intraoperatively collected information from MRI [5], CT, O-arm [4, 6], ultrasound (US) [7, 12] and 3D C-arm [1, 15] can also be used.

When a patient has foreign metal objects anywhere in the body, either known to be magnetic or of unknown magnetic properties, MRI is not allowed owing to the risk of severe damage due to possible movement or heating of the objects in the magnetic field [2]. Navigation would still be beneficial when removing these objects; therefore, other navigation imaging methods should be used for these purposes. A CT can be used, but unless there is a CT scanner available in the OR, it should be done preoperatively. The benefit of intraoperative imaging is that it can avoid errors from, e.g. brain shift, and give a real-time image to use in navigation. In paediatric cases, the use of intraoperative imaging also decreases the frequency of general anaesthesia (GA), since there is no need for preoperative imaging that is usually performed under GA.

O-arm is a cone-beam CT that can produce 2D and 3D images, which can be further used in neuronavigation. It is routinely used in navigation in spine surgeries. It can also be used in cranial stereotactic surgeries in selected cases [6]. The accuracy of O-arm is comparable to MRI navigation accuracy [8, 9]. Radiation dose is usually a bit larger than with fluoroscopy images with C-arm, but still distinctly smaller than with CT [13].

We report our experience of performing O-arm imaging-assisted neuronavigation during craniotomy in removing a bullet from the intracranial space in a paediatric patient.

## Case report

### Patient

The patient was a 14-year-old refugee boy from Syria who had been playing outdoors in his hometown at the age of 10 years. A battle begun during his play, and after the cessation of fire, he was found lying on the street unconscious. After admission to the local hospital, a low-resolution CT scan was taken. A bullet was seen intracranially close to the inner surface of the bone in the area of the sinus confluence. Due to high complication risks, it was decided not to remove the bullet. The patient gained consciousness within a couple of days and was discharged in approximately 10 days. Thereafter, the patient’s family sought their way to the refugee camp in Turkey and 3 years later to Finland.

**Fig. 1:**
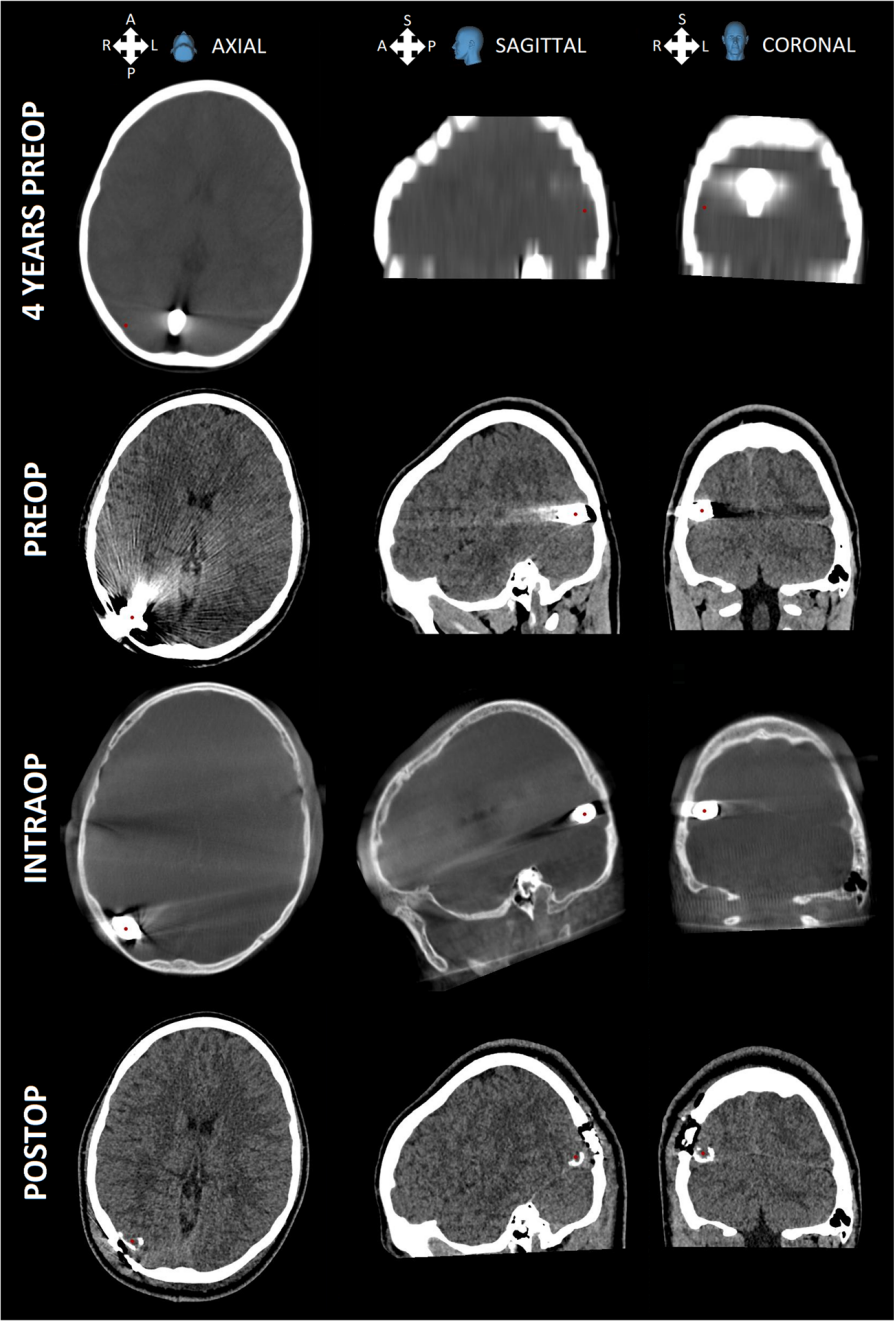
Upmost are the first CT images taken 4 years before surgery. Below that are CT images taken 4 months before surgery showing the bullet movement from midline to more lateral position. Next, intraoperative O-arm images present the situation at the time of surgery, and, finally, postoperative CT images reveal the calcified residual of the bullet’s capsule. Images are fused together with Medtronic StealthStation S8 and a red dot is placed in the centre of the bullet

In Finland, the patient suffered from headaches and mental health problems. He had short-lasting headache attacks several times a week ever since the accident. The episodes usually lasted 5–20 min with pain starting in the occipital region and radiating behind the right eye. During these headaches, the patient usually went supine due to the devastating pain. In clinical investigation, no neurological deficiencies were found. Neither were there any abnormal findings on skull palpation. On ophthalmological investigation, myopia, requiring correction with glasses, as well as borderline reduction of an upper medial visual field were found. The later finding was interpreted as a possible result of the damage to the occipital cortex. A 3D skull CT scan was performed. According to the scan, the bullet seemed to be preserved in one piece and located close to the inner surface of the skull bone near the lambdoid suture, in contact with the tentorium just above the sinus transversus. Compared to CT scans 4 years earlier, the bullet had moved approximately 29 mm laterally (Fig. 1).

The patient and his parents wished the bullet to be removed because of the pain located precisely at the bullet area, in the hope of headache cessation after successful surgery. The decision to remove the bullet, in addition to the wishes of the patient and his parents, was based on the following considerations. At first, the removal of the ferromagnetic foreign object would allow performing MR imaging whenever needed. Second, the bullet had already moved almost 3 cm away from primary location during the follow-up time, and further dislocation of the bullet risked causing new damage. Third, the relatively superficial location of the bullet supported the decision to proceed with operative treatment.

Prior to the operation, up-to-date images of the bullet’s location and vascular structures in its proximity were necessary. The 3D CT scan done previously in a central hospital was not suitable for navigation due to slice thickness (Fig. 1). In addition, preoperative images revealed the movement of the bullet, leading to the decision to use neuronavigation with intraoperative O-arm imaging and automatic direct registration to the navigation system.

### Surgical imaging and navigation

We used the StealthStation S7 (Medtronic Inc., Louisville, CO, USA) with optical localization method for navigation. Due to the lack of O-arm navigation implementation in Cranial software, we used the Spine software to allow an automated registration of the O-arm images. 

The intraoperative imaging was done with O-arm (Medtronic Inc., Louisville, CO, USA) and 3D images (slice thickness was 0.833 mm, tube voltage 100 kV, tube current 80 mA and imaging time 13 s) were automatically transferred into navigation system.

### Surgical procedure

**Fig. 2:**
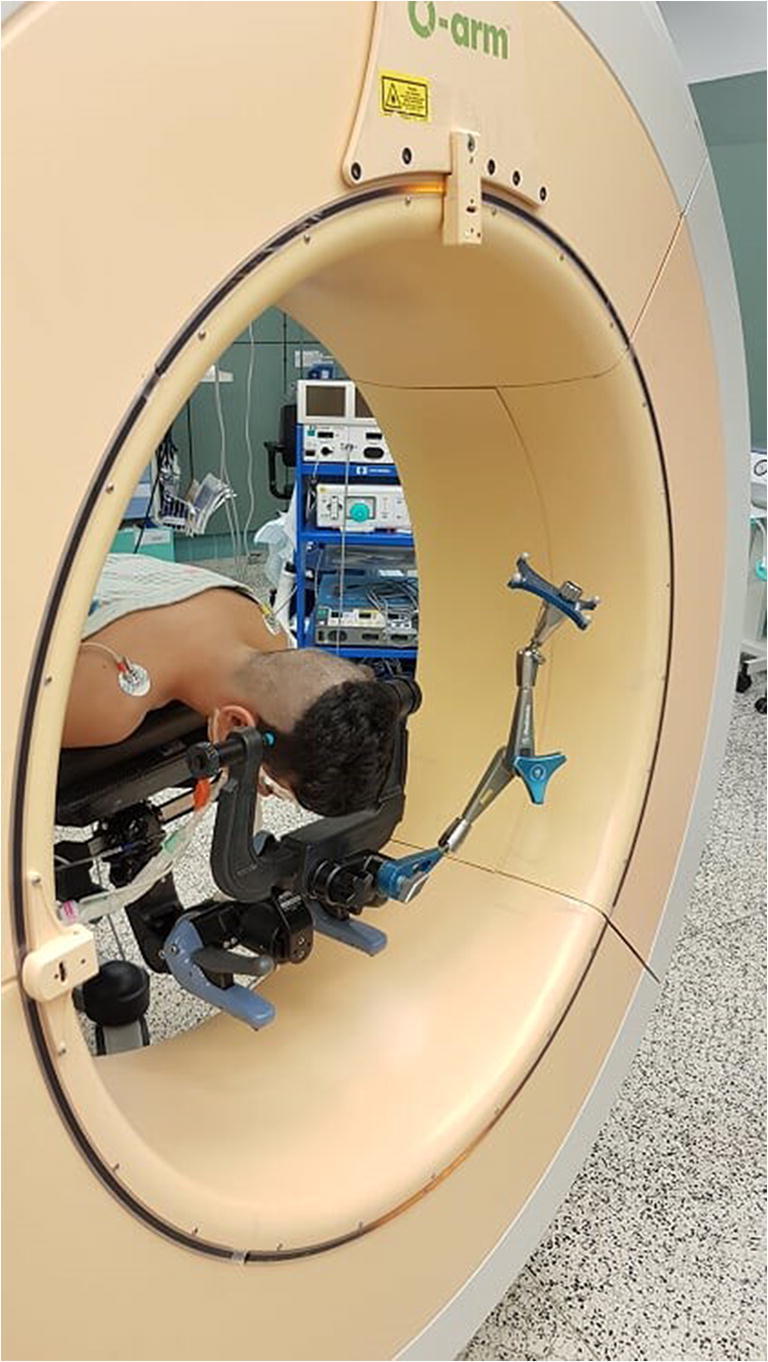
The patient, the O-arm and the navigation system positioning for the surgery

**Fig. 3:**
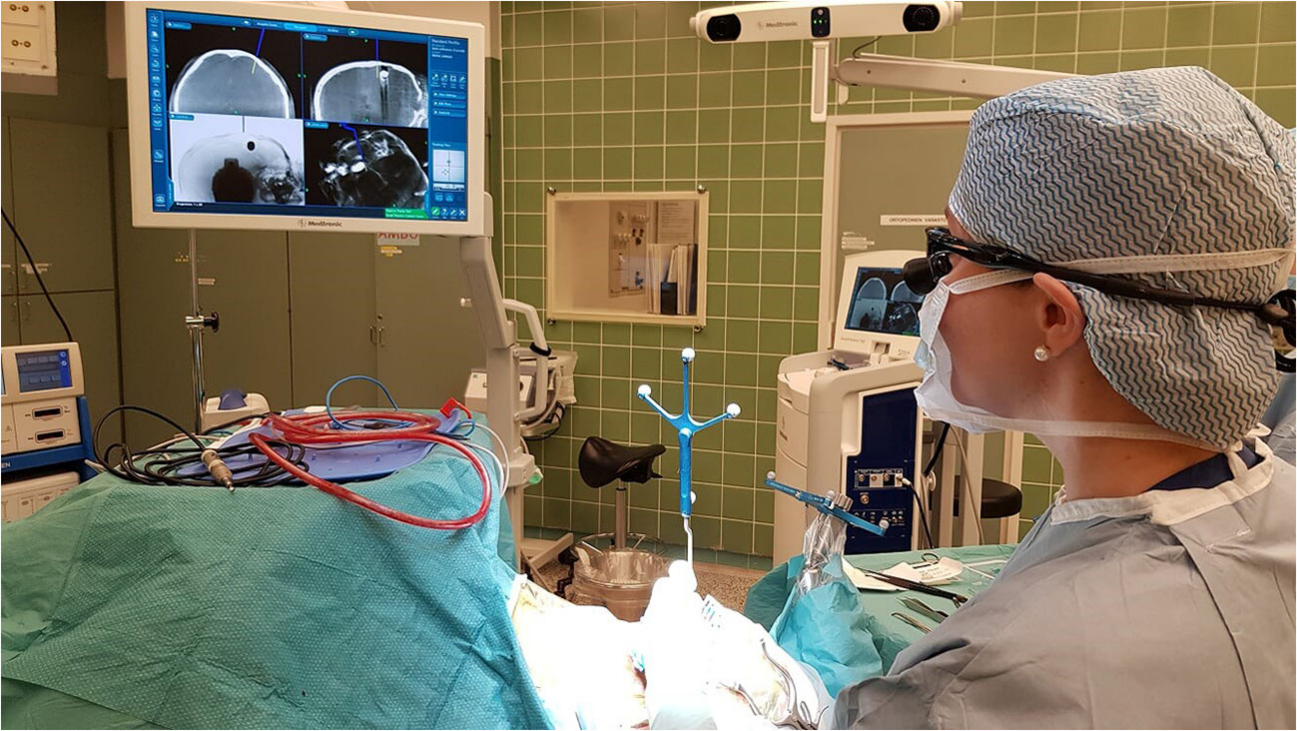
The operation room setup during the procedure. Surgeon navigating the opening point

**Fig. 4:**
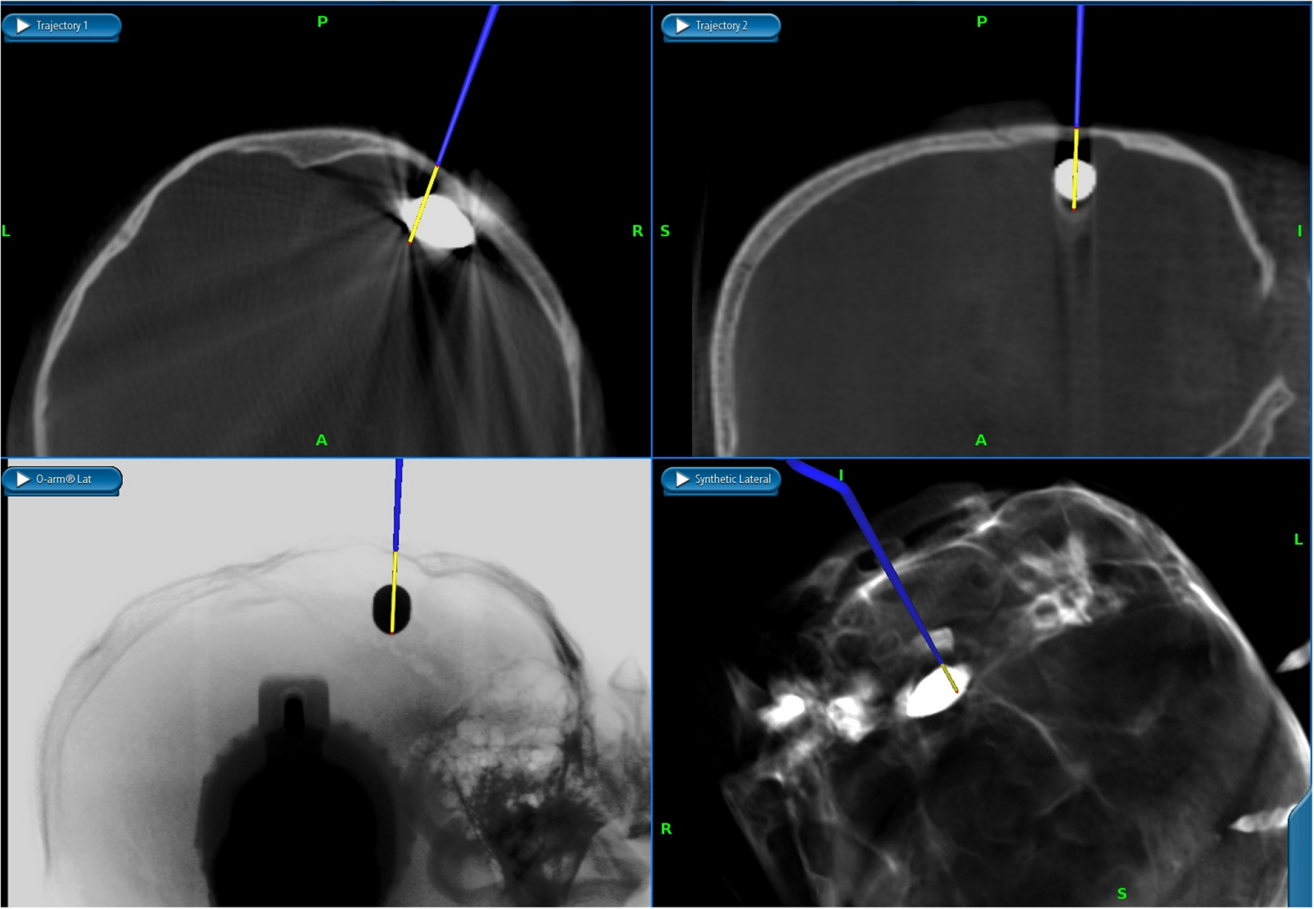
The navigation view in StealthStation 7 Spine software. Upper row shows the trajectories perpendicular to the navigation pointer

**Fig. 5:**
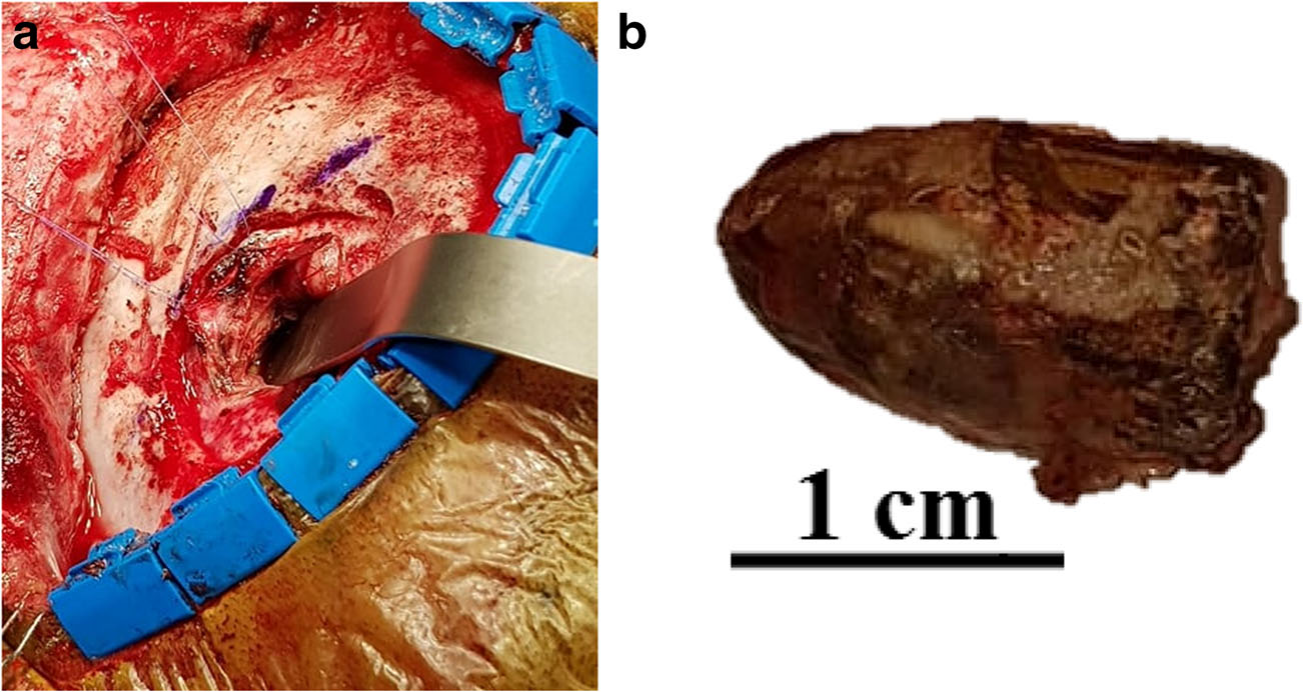
**a** Intraoperative view after craniotomy with the spathel pointing at the bullet. **b** The removed bullet with 1-cm scale next to it

The patient was set in a prone position with his head fixated using the carbon fibre frame (Fig. 2). After the O-arm imaging and navigation (Fig. 3), an L-shaped skin opening was made in the midline extending to the right side. After opening of the soft tissues, the lambdoid suture and the bullet were located using navigation (Fig. 4). Craniotomy was done in a conventional way with its lower line going just above the sinus transversus edge. The dura was preserved intact during the bone removal and its appearance was normal. After opening of the dura the encapsulated bullet was found lying on the surface of an apparently normal-looking cortex. The capsule was opened sharply, and the bullet was removed (Fig. 5). The capsule wall was calcified, and it was adherent to the venous sinus wall and to the cortex. For this reason, it was decided not to remove this  fibrotic capsule. The dura was sutured with continuous suture. Hemocoagulate patch (Tachosil, Takeda Austria GmbH, Austria) and tissue glue (Evicel, Omrix Biopharmaceuticals Ltd., Israel) were used to ensure watertight closure. The bone was fixated with Matrix low-profile titanium alloy plates and screws. The skin was closed in a conventional way.

Postoperative testing of the removed bullet showed that it was not ferromagnetic. The bullet appeared to be a 9 × 19-mm Parabellum pistol cartridge (Fig. 5b). The calcified residual of the capsule was seen in the postoperative CT images. The postoperative CT showed no signs of any complications. The recovery after surgery was uneventful and the patient was discharged on the 5th postoperative day. After 1 year of follow-up, the patient had made a full recovery and had neither headaches nor any other symptoms.

## Discussion

Removing an intracranial foreign body is a surgical procedure that normally involves a high risk of complications. In cases in which the patient is a child, the surgery-related sequelae may be still more important, taking the long life expectancy into consideration. For these reasons, as mini-invasive, accurate and safe surgical procedure as possible is appreciated in removing any intracranial foreign object . The reported technique was feasible when using navigation without preoperative MRI. Knowing the precise location of the bullet from the very beginning of the surgery allowed for minimizing soft tissue damage and shortening the duration of the procedure. Both the short- and long-term outcomes were good and the patient recovered without any complications.

In gunshot injuries of paediatric patients, early surgery is recommended, as it is assumed to increase the survival rate and decrease postoperative complications [10]. In contrast, there is no agreement regarding the optimal treatment strategy in patients with a long history of an intracranial foreign material. In this case, the bullet had been in the brain more than 3 years and one option would have been to leave the bullet in place. However, the removal of the bullet was evaluated to be a preferred choice as there was a risk of further movement of this object. In addition, the removal of the foreign material would enable undertaking any MRI the patient might need in the future. The patient also suffered from strong headaches, which were associated with the injury. Due to non-existing mass effect, and no signs of any inflammation in the area of the bullet, we doubt that the headaches were related to the brain injury or posttraumatic psychological reactions related to the primary injury.

The surgical procedure was performed without any primary complications. Due to minimized soft tissue damage and minimal opening during the surgery, the patient stayed just 5 days in the hospital after the surgery and made a fast recovery, only requiring strong pain medication during the first postoperative days. The radiation dose of the patient in this O-arm navigation was evaluated to be reasonable compared to all the benefits this method permitted.

Furthermore, there was additional advantage of the reported new technique, as compared with the other potential treatment methods: the radiation dose for the personnel in this surgery was very minimal. There was only one person in the operation room during the O-arm imaging wearing conventional radiation protection clothing and standing behind the movable radiation protection wall during imaging. 

Foreign body injuries in children are not unusual and they typically concern the natural cavities of the body [14]. Foreign objects in a tissue, such as the brain, have been rare in developed countries. However, there has been increasing migration recently, and potentially more severe intraparenchymal foreign body injuries, e.g. as a consequence of a shooting incident or war, are no longer a simply rare event. Therefore, the inability to use MRI for navigation is a growing problem as immigrants from military areas can have, e.g. retained bullets from firearm injuries or fragments of missiles/bomb shells. The ferromagnetic metal objects can rotate and/or move in a magnetic field and thus possibly result in secondary damage [2, 3, 11]. 

In this report, we demonstrated a sophisticated surgical method in treating a paediatric patient who suffered from a metallic foreign body in his brain. The O-arm was proven to be an accurate method, which is in agreement with previous understanding [8, 9]. Another benefit of the method is the ability to use the automatic registration of intraoperative images to the navigation system. The surgical method was described in detail and the procedure can be replicated in other institutions. However, the study is open to criticism as it is limited to only one case and the experience of the method is therefore not wide. Greater series are recommended to get more evidence about this innovative technique in the future. Due to a risk of vascular damage or possible contamination caused by the bullet, when treating similar cases, a preoperative angiography and itnraoperative microbiological swaps with prolonged prophylactic antibiotic treatment should be considered.

## Conclusion

The O-arm was successfully used in navigation, when an intracranial bullet was removed from a paediatric patient. Compared to the traditional surgical approach without any perioperative imaging, the method improved the accuracy of the procedure by localizing the bullet, the veins and the fibrotic capsule in close proximity.
